# Genomic Insights into Myasthenia Gravis Identify Distinct Immunological Mechanisms in Early and Late Onset Disease

**DOI:** 10.1002/ana.26169

**Published:** 2021-08-04

**Authors:** Lahiru Handunnetthi, Bogdan Knezevic, Silva Kasela, Katie L. Burnham, Lili Milani, Sarosh R. Irani, Hai Fang, Julian C. Knight

**Affiliations:** ^1^ Wellcome Centre for Human Genetics University of Oxford Oxford UK; ^2^ Nuffield Department of Clinical Neurosciences University of Oxford Oxford UK; ^3^ Estonian Genome Centre, Institute of Genomics University of Tartu Tartu Estonia; ^4^ Wellcome Sanger Institute Cambridge UK

## Abstract

**Objective:**

The purpose of this study was to identify disease relevant genes and explore underlying immunological mechanisms that contribute to early and late onset forms of myasthenia gravis.

**Methods:**

We used a novel genomic methodology to integrate genomewide association study (GWAS) findings in myasthenia gravis with cell‐type specific information, such as gene expression patterns and promotor‐enhancer interactions, in order to identify disease‐relevant genes. Subsequently, we conducted additional genomic investigations, including an expression quantitative analysis of 313 healthy people to provide mechanistic insights.

**Results:**

We identified several genes that were specifically linked to early onset myasthenia gravis including *TNIP1*, *ORMDL3*, *GSDMB*, and *TRAF3*. We showed that regulators of toll‐like receptor 4 signaling were enriched among these early onset disease genes (fold enrichment = 3.85, *p* = 6.4 × 10^−3^). In contrast, T‐cell regulators *CD28* and *CTLA4* were exclusively linked to late onset disease. We identified 2 causal genetic variants (rs231770 and rs231735; posterior probability = 0.98 and 0.91) near the *CTLA4* gene. Subsequently, we demonstrated that these causal variants result in low expression of *CTLA4* (rho = −0.66, *p* = 1.28 × 10^−38^ and rho = −0.52, *p* = 7.01 × 10^−22^, for rs231735 and rs231770, respectively).

**Interpretation:**

The disease‐relevant genes identified in this study are a unique resource for many disciplines, including clinicians, scientists, and the pharmaceutical industry. The distinct immunological pathways linked to early and late onset myasthenia gravis carry important implications for drug repurposing opportunities and for future studies of drug development. ANN NEUROL 2021;90:455–463

Myasthenia gravis is an autoimmune disease that manifests clinically as muscle weakness and fatigability. Approximately 80% of patients with generalized myasthenia gravis have autoantibodies against the acetylcholine receptor (AChR) in the post‐synaptic muscle endplate.^1^ Interestingly, these patients with AChR antibodies have a bimodal pattern of disease onset with an early peak around 30 years of age and a late peak around 70 years of age.^1^, ^2^ The early onset (EO) and late onset (LO) forms of myasthenia gravis have distinct epidemiological and pathological characteristics.^1^ The majority of EO patients have thymic hyperplasia characterized by germinal centers, whereas these features are not common in LO patients.^3^ Thymoma associated myasthenia gravis is another distinct group and nearly all of these patients have detectable AChR antibodies and generalized disease.^1^ The mechanisms underlying these observations remain unresolved but divergent immunological pathways have been proposed to account for the differences between EO and LO forms of myasthenia gravis.

Recent genomewide association studies (GWAS) have uncovered a number of genetic risk loci in myasthenia gravis providing a glimpse into the underlying disease mechanisms.^4^, ^5^, ^6^ These studies suggested that distinct genetic risk factors could contribute to the pathogenesis of EO and LO myasthenia gravis. Notably, different genetic variants within the major histocompatibility complex (MHC) locus contribute to EO and LO disease.^4^, ^5^, ^6^ Several risk loci outside the MHC have been identified through GWAS; for example, genetic risk variants near the *TNIP1* gene were linked to EO disease^4^ whereas those close to the *TNFRSF11A* gene were associated with LO disease.^6^ Despite this initial success, GWAS in myasthenia gravis were relatively underpowered and most risk variants fell into noncoding regions. Therefore, much of the genetic susceptibility to myasthenia gravis remains to be elucidated.

It is important to note that each genetic risk locus identified through GWAS contains many plausible disease relevant genes and further work is needed to identify the genes that contribute to the causal cascade. The emerging evidence suggest that causal genetic variants are likely to exert functional effects through changes to gene expression, often in a cell‐type‐specific manner.^7^ Furthermore, these causal variants may modulate the expression of distant genes through chromatin looping.^8^ We recently developed Priority Index, a genomic methodology that integrates cell‐type‐specific functional genomic information, such as gene expression and chromatin organization, with GWAS findings to identify disease relevant genes.^9^ In this study, we build on our previous work and apply Priority Index to myasthenia gravis GWAS results in order to identify disease relevant genes, and to specifically explore differences between EO and LO myasthenia gravis disease.

## Materials and Methods

### Study Samples

We included data from 3 published GWAS investigating patients with AChR antibody positive myasthenia gravis.^4^, ^5^, ^6^ The study characteristics and main findings of each GWAS are summarized in Table 1. All myasthenia gravis associated single nucleotide polymorphisms (SNPs) achieving genomewide *p* value of <5 × 10^−5^ (and co‐inherited genetic variants based on linkage disequilibrium of r^2^ > 0.9) were included. We excluded the MHC from our analyses because of its high linkage disequilibrium. Health Research Authority (HRA) approval was not required for this work because only publicly available data were used. This work was subject to the Oxford University Research Integrity and Ethics Policy.

**TABLE 1:** Study Characteristics and Findings from Genome Wide Associations Studies in Myasthenia Gravis

Study	Study size	Patient characteristics	Main findings
Renton et al 2015	Overall: 1032 patients 1998 controls EO: 235 patients 1,977 controls LO: 737 patients 1,977 controls	AChR Ab +ve EO and LO patients Age: EO age <40 years LO age >40 years Sex: 694 female patients (47.4%)	LO findings: MHC class II and one non‐MHC risk locus (lead SNP: rs4263037 and suggested gene *TNFRSF11A*) as well as other suggestive risk loci. EO findings: MHC class II and several suggestive non‐MHC risk loci Combined: MHC class II Two non‐MHC risk loci: Chr 18 (lead SNP: rs4263037, suggested gene *TNFRSF11A*) and Chr 2 (lead SNP: rs231770, suggested gene *CTLA4*)
Seldin et al 2015	532 patients 2,128 controls	AChR Ab +ve non‐thymomatous LO patients Age: ≥50 years Sex: 200 (37.6%) female patients	MHC associations with HLA‐A, class II and III One non‐MHC risk locus on Chr 8 (lead SNP: rs6998967, suggested gene *ZBTB10*) and several other suggestive risk loci
Gregersen et al 2012	649 patients 2,596 controls	AChR Ab +ve non‐thymomatous EO patients Age: <40 years or <45 years with hyperplastic thymic histology Sex: 538 (82.9%) female	MHC association with HLA‐B*08 Two non‐MHC risk loci on Chr 1 (lead SNP: rs2476601, suggested gene *PTPN22*) and Chr 5 (lead SNP: rs4958881, suggested gene *TNIP1*) as well as several other suggestive risk loci.

### Priority Index for Gene Prioritization

We integrated disease associated SNPs with cell‐type‐specific genomic information to identify genes relevant to EO and LO myasthenia gravis. Specifically, we tested if myasthenia gravis associated SNPs were located in regions of enhancer‐promoter interactions (chromatin conformation capture)^8^ and were linked to gene expression changes (expression quantitative trait loci)^10^, ^11^, ^12^, ^13^ in immune cell types (B cells, CD4^+^ T cells, CD8^+^ T cells, NK cells, and monocytes). This allowed us to identify likely causal genes in the disease. We identified additional genes relevant to myasthenia gravis based on their network connectivity to the likely causal genes. Subsequent quantification of this genomic evidence and network connectivity produced ranked lists of disease relevant genes for EO and LO disease. We also carried out a combined analysis irrespective of the timing of disease onset. Gene prioritization was carried out using the bioinformatics pipeline Priority Index.^9^ Customizable options, such as inclusion of genes associated with rare diseases and/or various human and mouse phenotypes in Priority Index pipeline, were omitted in order to ensure our gene prioritization was entirely based on GWAS results. Software and coding information relating to Priority Index is available at (http://bioconductor.org/packages/Pi) and more information about the application and validity of methodology is available from Fang et al 2019.^9^

### Gene Set Enrichment Analyses

Gene enrichment analyses provide biological insight into disease relevant genes by assessing if these genes are over or under‐represented among well‐characterized gene sets, such as members of specific biological processes. Accordingly, we tested if genes with evidence for their role in EO and LO disease, identified through Priority Index were over‐represented among informative gene sets including biological pathways from the Reactome Knowledgebase^14^ as well as those genes linked to viral infections,^15^ TLR signaling,^16^ and active disease status in patients with myasthenia gravis.^16^ These enrichment tests were conducted using the *xEnricher* function in the R package “Pi” (version 1.5.1) implementing a hypergeometric test of significance without replacement at *p* < 0.05 significance threshold. Further, ranking of genes relevant to EO and LO disease was taken into account in gene set enrichment analyses where applicable. Gene set enrichment was quantified from 3 different features, (1) enrichment change – the normalized enrichment score (NES) calculated as the observed running enrichment score (ES) divided by the expected; (2) enrichment significance – the *p* value; and (3) enrichment coverage – the fraction of genes found at the “leading region” (defined as genes that appear at or before the running enrichment score reaches its maximum deviation from zero). The expected ES was estimated according to a null distribution generated by randomly sampling gene sets 20,000 times.

### Fine‐Mapping the *CTLA4* Locus

We carried out a detailed interrogation CTLA4 risk locus in myasthenia gravis to identify the likely disease causal genetic variants. This was achieved by integrating the strength of GWAS associated SNPs with annotations of gene regulatory elements within this risk locus. Specifically, we used a collection of histone modifications H3K27ac and H3K4me1 (indicative of enhancers), H3K27me3 (transcription repressors), and H3K4me3 (promoters) as well as DNase I hypersensitivity sites (accessible chromatin related to transcriptional activity)^17^ from CD4+ T cells as our annotators. This fine mapping was carried using Probabilistic Annotation INtegraTOR (PAINTOR)^18^ and the disease associated SNPs for fine‐mapping were sourced from the Renton et al 2015 study.^5^

### Expression Quantitative Trait Analysis

We next sought to investigate if the likely causal variants in the *CTLA4* risk locus can exert functional effects on gene expression. In order to achieve this, we tested if the fine‐mapped genetic variants colocalized with eQTLs identified in CD4+ T cells. Identification of cis‐eQTLs in CD4+ T cells was described in our previously published work.^10^ Briefly, CD4^+^ T cells were purified from peripheral blood mononuclear cells in 313 healthy European individuals. This included 154 women and 159 men with a median age of 54 years (standard deviation = 17.8). Genomewide gene expression profiling and genotyping were performed using HumanHT‐12 version 4 BeadChips (Illumina) and HumanOmniExpress‐12 version 1.0 BeadChips (Illumina), respectively. Following quality control and filtering, around 6 million SNPs and expression from 38,839 probes in 23,704 genes were included in the analysis. Cis‐expression quantitative trait locus (eQTL) calculation was conducted by testing for association between SNPs and gene expression within 1 Mb intervals. Colocalization of signal between eQTL and myasthenia GWAS was carried out using Coloc.^19^ This method estimates the posterior probability that the SNPs in question are causal in both GWAS and eQTL studies.

## Results

### Distinct Gene Networks in Early and Late Onset Myasthenia Gravis Disease

**FIGURE 1:**
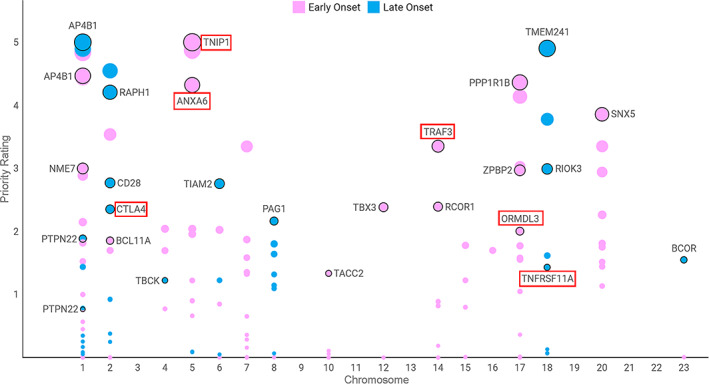
Disease relevant genes in early and late onset myasthenia gravis. Prioritized genes with direct genomic evidence based on proximity to myasthenia gravis associated SNPs accounting for linkage disequilibrium (nGene), physical interaction between myasthenia gravis associated SNPs and enhancer‐promoter regions based on chromatin conformation (cGene) and modulation of gene expression based on expression quantitative trait mapping (eGene) in major immune cell types. The y‐axis shows the gene priority rating and the x‐axis shows their chromosome position. The diameter of the circle represents the ‐log10 (*p*‐value) and boxes represent genes with multiple layers of genomic evidence in myasthenia gravis. [Color figure can be viewed at www.annalsofneurology.org]

Several disease relevant genes were identified for EO and LO myasthenia gravis using Priority Index (Fig 1; Supplementary Tables [Link], [Link]). We identified novel genes *ORMDL3*, *GSDMB*, and *TRAF3* that exclusively contribute to EO disease. We also found genomic evidence for the previously implicated *TNIP1* in EO disease. On the other hand, we discovered genomic evidence for several novel genes including *BCOR* and *CD28* as well as for previously suggested gene *CTLA4* in LO disease. Furthermore, we found that 26 genes were shared in the top 1% of disease relevant genes between EO and LO myasthenia gravis (see Supplementary Table S5).

We hypothesized that disease relevant genes identified from the combined analysis of EO and LO associated SNPs would overlap with the gene expression signature of patients with myasthenia gravis. We carried out gene set enrichment analyses using data from a published blood transcriptomic study of active versus remission‐state myasthenia gravis in order to test this hypothesis.^16^ We found that the top 1% of the disease relevant genes identified through our genomic approach were significantly enriched among the differentially expressed genes in the active disease state more than expected by chance (*p* = 9.5 × 10^−3^). Moreover, 5 out of 7 genes in the leading edge of our gene set enrichment analysis were previously identified as key candidates (*S100P*, *GAB2*, *NFKBIA*, *TNFAIP3*, and *PPP1R15A*) for molecular signatures of disease activity in myasthenia gravis, highlighting the validity of our independently prioritized genes from GWAS results.

### Causal Variants Are Associated with Low *CTLA4* Expression

**FIGURE 2:**
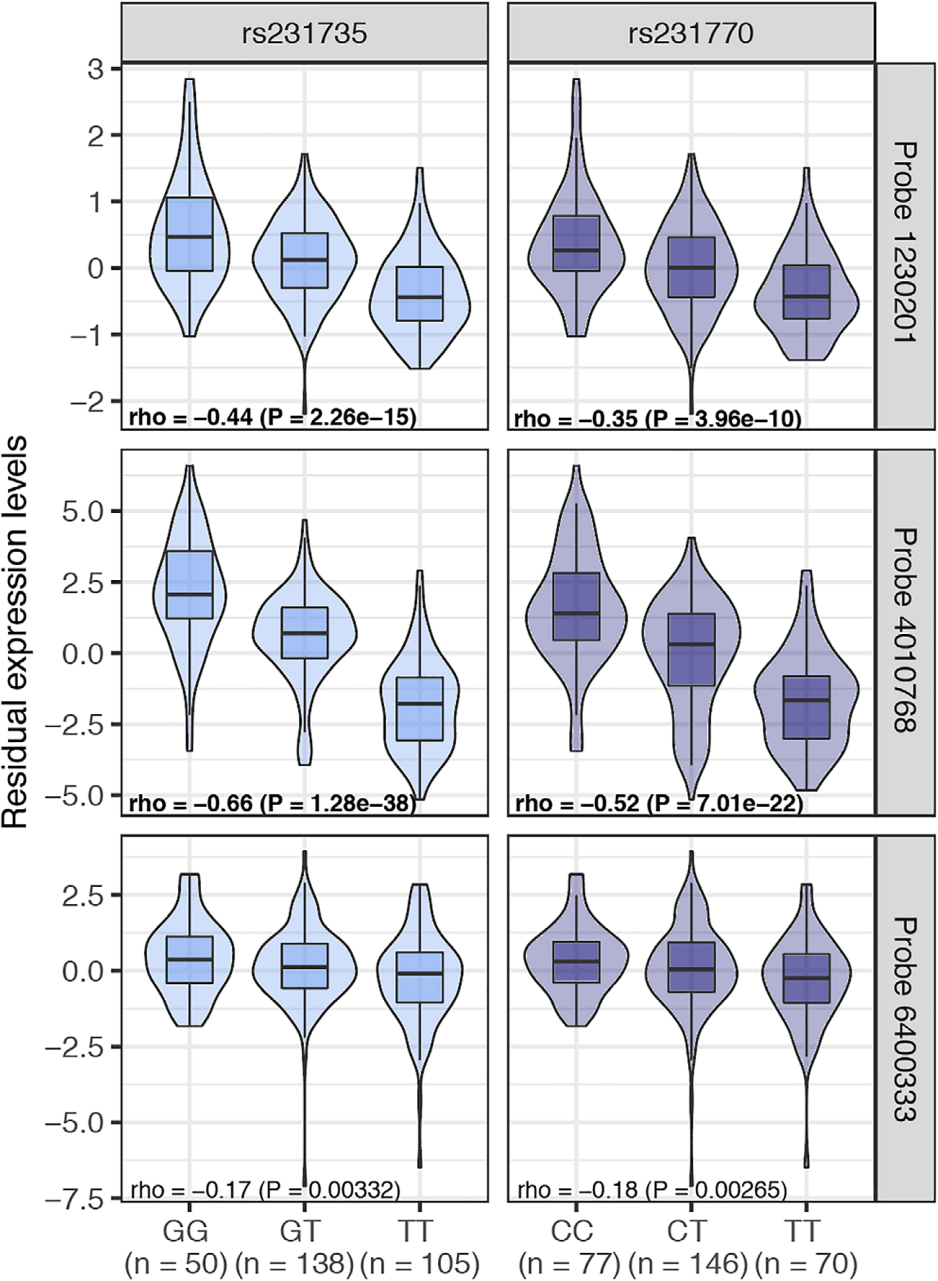
CTLA4 locus and myasthenia gravis Violin plots showing the allelic effect of likely causal SNPs (x‐axis) on the expression levels in CD4+ cells (yaxis) for three different probes corresponding to CTLA4 gene. The lower and upper border of the box correspond to the first and third quartiles, respectively, the central line depicts the median, and whiskers extends from the borders to ±1.5xInter‐quantile range. Significant eQTLs at FDR 5% are highlighted in bold. [Color figure can be viewed at www.annalsofneurology.org]

We next focused on the LO associated gene *CTLA4*. This gene is of particular interest because immune check point inhibitors that target *CTLA4* in the treatment of metastatic melanoma can lead to the development of myasthenia gravis.^20^, ^21^ We identified 2 disease associated SNPs (rs231770 and rs231735) with high posterior probability of causality within the *CTLA4* locus (0.98 and 0.91, respectively). These SNPs overlapped with gene promoter and enhancer elements revealing a potential disease mechanism through altered gene expression of *CTLA4* in LO myasthenia gravis disease. We then investigated whether these 2 likely causal SNPs could influence the expression of *CTLA4* in an eQTL analysis of CD4^+^ T cells in 313 healthy individuals. We found that both SNPs were associated with the expression of *CTLA4*. Probe 4010767 was the most significant (rho = −0.66, *p* = 1.28 × 10^−38^ and rho = −0.52, *p* = 7.01 × 10^−22^, for rs231735 and rs231770, respectively, both meeting a false discovery rate [FDR] < 0.05; Fig 2). The minor alleles matched the GWAS identified risk alleles and were associated with lower *CTLA4* gene expression. Further colocalization analysis confirmed that there was a high likelihood of shared causal signal between the eQTL and GWAS datasets (posterior probability of 0.925).

### Innate Immune Pathways Are Exclusively Linked to Early Onset Myasthenia Gravis

**FIGURE 3:**
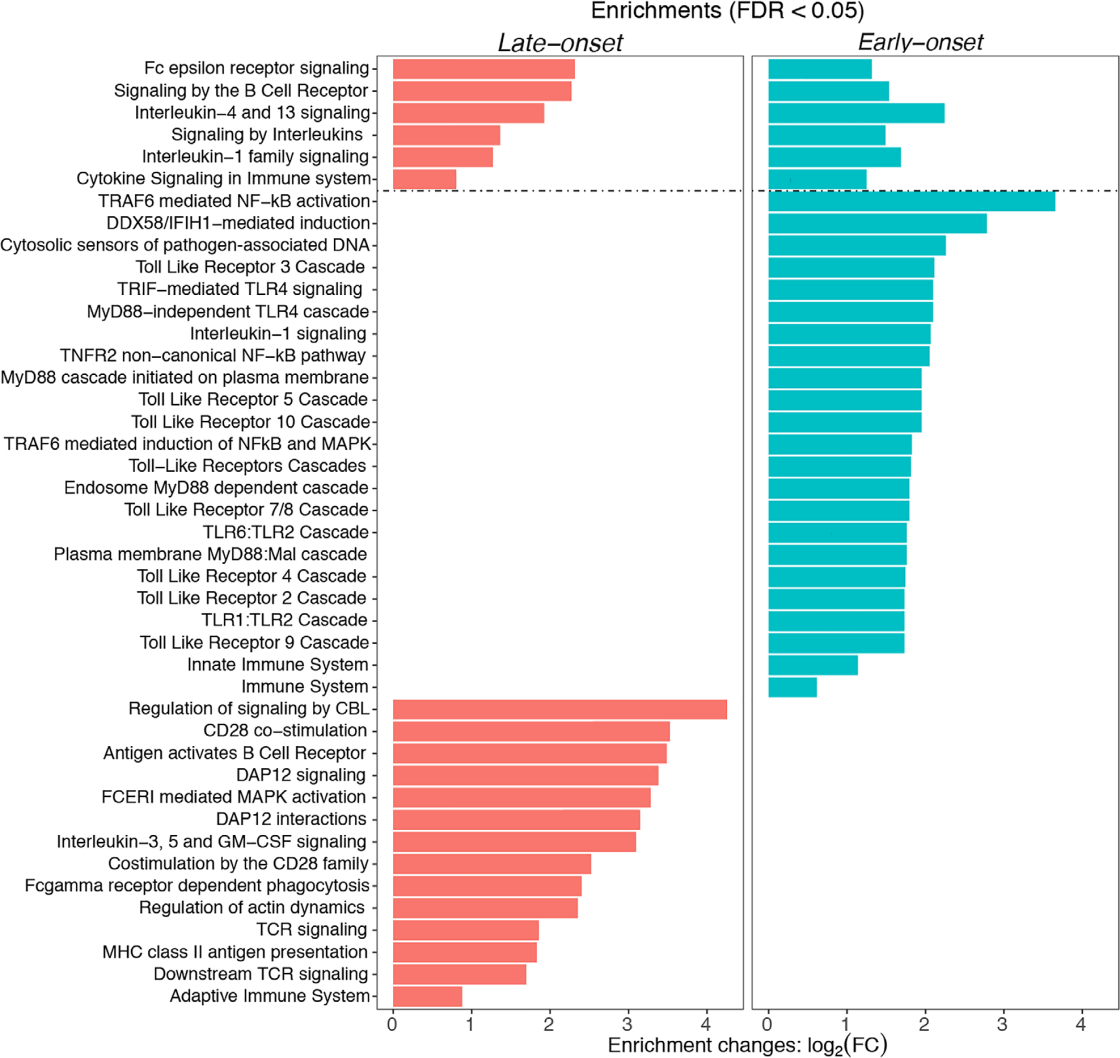
Immune pathways in early and late onset myasthenia gravis. Overview of prioritized Reactome Immune System Pathways based on the top 1% of disease relevant genes in EO and LO form of myasthenia gravis. This highlights both shared and unique pathways between EO and LO disease. The x‐axis is log‐transformed fold‐change (FC) in enrichment. All pathways presented met a FDR < 0.05 cutoff. [Color figure can be viewed at www.annalsofneurology.org]

We next conducted a pathway analysis of the top 1% genes in EO and LO disease using the REACTOME database. The results highlighted several key overlapping and unique immunological pathways between EO and LO myasthenia gravis (Fig 3). We found that B‐cell receptor signaling pathways (EO fold change = 2.90, FDR = 2.4 × 10^−2^; LO fold change = 4.83, FDR = 1.6 × 10^−3^) were highly enriched in both forms of the disease. We found that EO genes were exclusively enriched among innate immune recognition pathways for pathogens, such as TRAF6‐mediated NFkB activation (fold change = 12.6, FDR = 1.4 × 10^−3^), viral sensing through DDX58/IFIH (fold change = 6.89, FDR = 1.4 × 10^−3^), and toll‐like receptor (TLR) 4 signaling (fold change = 4.28, FDR = 8.3 × 10^−3^). In contrast, LO pathways were related to T‐cell function, such as CD28 co‐stimulation (fold change = 11.5, FDR = 2.9 × 10^−4^) and MHC class II presentation (fold change = 3.55, FDR = 8.4 × 10^−3^).

We performed additional analyses using independently generated genomic data to further interrogate the disease onset specific differences. First, we investigated how the prioritized genes in EO and LO disease captured known regulators of Tlr4‐mediated TNF induction, identified through a genomewide CRISPR‐Cas9 screen.^22^ We found that the top 10% of EO prioritized genes were significantly enriched for these regulators (3.85‐fold enrichment, *p* = 6.4 × 10^−3^), while LO genes were not (*p* = 0.746). Moreover, a leading‐edge analysis of Tlr4 regulators and the EO genes recovered 51 out of 81 targets (*p* = 2.0 × 10^−3^). Second, we used viral infection related gene signatures^15^ to show that EO genes were significantly enriched for viral infection associated genes (*p* = 0.019) as well as Epstein–Barr Virus (EBV) unique genes (*p* = 0.041), whereas LO genes were not (*p* = 0.117 and *p* = 0.274, respectively).

## Discussion

In this study, we integrated GWAS findings from patients with AChR antibody positive myasthenia gravis with cell‐type specific genomic information in immune cells to identify disease relevant genes. Importantly, our results highlight several key differences in the immunological pathways between EO and LO disease and support the notion that multiple mechanisms can lead to autoimmunity in myasthenia gravis. Findings from this study advance the knowledge gained through GWAS by providing functional evidence for previously implicated genes, such as *TNIP1* and *CLTA4* in EO and LO myasthenia gravis, respectively.^4^, ^5^ Both *TNIP1* and *CLTA4* have previously been linked to other autoimmune diseases providing credence to our findings.^23^, ^24^ In addition, we provide support for several novel genes, including *TRAF3*, *ORMDL3*, and *GSDMB* that contribute exclusively to EO disease. *TRAF3* is known to regulate both innate and adaptive immunity^25^ and its deficiency leads to lymphoid organ disorders and autoimmune manifestations.^26^ Further, *ORMDL3* plays an important role in B‐cell survival^27^ and genetic variants associated with other immune‐mediated diseases have previously been shown to influence the expression of *ORMDL3* and *GSDMB* on chromosome 17q21.^28^, ^29^

We identified a unique role for the innate immune system in EO myasthenia gravis disease. First, several EO genes, such as *TRAF3* and *TNIP1*, identified in this study are key regulators of TLR signaling.^24^, ^25^ Second, we found that known regulators of Tlr4‐mediated TNF induction were exclusively enriched among EO genes. This complements the previously observed aberrant TLR4 signaling in the hyperplastic thymuses of patients with myasthenia gravis.^30^ Third, we showed that gene expression changes related to common viral infections and those changes unique to EBV infection were exclusively enriched among EO genes. Our results suggest common viral infections could contribute to EO disease but the role of EBV in myasthenia gravis disease is debated.^31^, ^32^ Collectively, our findings add to a growing body of evidence that the innate immune system plays a key role in driving the pathogenic antibody production in EO myasthenia gravis disease.^33^ Therefore, targeting of TLR signaling could represent a new therapeutic avenue for EO disease and warrants further investigation.

We identified multiple layers of evidence that T‐cell pathways are important in the pathogenesis of LO myasthenia gravis disease. We found that *CD28* and *CTLA4* contribute exclusively to LO disease. Together CD28 and CTLA‐4 regulate T‐cell activation and proliferation^34^ and their roles in autoimmunity are well studied.^22^, ^35^ We fine mapped the *CLTA4* locus in myasthenia gravis and demonstrated for the first time that causal alleles result in low expression of *CTLA4* in CD4+ T‐cells. These findings complement the clinical features associated with CTLA‐4 deficiency in humans,^36^ and previous observations that CTLA‐4 deficient mice develop autoimmunity,^35^ as well as high soluble serum levels (ie, low cell surface expression) in patients with myasthenia gravis.^37^ It is not clear exactly how low levels of CTLA‐4 lead to the production of AChR antibodies but emerging evidence highlight a role for CTLA‐4 in both T‐regulatory cells in modulating humoral immunity.^38^ Interestingly, the CTLA‐4 fusion protein abatacept is used to treat CTLA‐4 insufficiency^39^ and this could represent a repurposing opportunity in LO myasthenia gravis disease. Indeed, those patients who are likely to respond to abatacept could be selected based on the causal genetic variants identified in this study.

This study was limited by the relatively underpowered GWAS in myasthenia gravis.^4^, ^5^, ^6^ In addition, we could only leverage cell‐type‐specific genomic information from immune cells. Future studies using as yet undiscovered GWAS hits and genomic information from other cell populations will reveal further information about disease mechanisms. Nonetheless, our results provide novel new insights into immune pathways associated with EO and LO disease. The apparent dichotomy between EO and LO disease supports the long‐held hypothesis that myasthenia gravis disease is a heterogeneous disease and multiple autoimmune mechanisms ultimately lead to the production of AChR antibodies.^40^ Based on our findings, we can hypothesize that innate and adaptive immune systems interact closely in the thymus to produce pathogenic antibodies in EO disease whereas LO disease may be mediated by loss of peripheral tolerance. These distinct genes and pathways could address unanswered research questions like why there is a preponderance of women in EO disease and carry important implications for personalized treatment strategies in the future. We envisage that our findings will accelerate the much needed development of targeted therapeutic options for EO and LO myasthenia gravis disease through genomics‐led drug discovery programs.

## Author Contributions

L.H., H.F., and J.C.K. contributed to conception and design of this study. L.H., B.K., and K.S. contributed to the acquisition and analysis of data. L.H., J.C.K., B.K., K.L.B., H.F., S.R.I., S.K., and L.M. contributed to drafting a significant portion of the manuscript or figures.

## Potential Conflict of Interests

No conflicts of interests to declare.

## Supporting information


**Table S1**. Genes with Direct Genomic Evience (seed genes) in Early Onset Myasthenia Gravis

**Table S2**. Genes with Direct Genomic Evience (seed genes) in Late Onset Myasthenia Gravis

**Table S3**. All Early Onset Genes (seed genes and non‐seed genes)

**Table S4**. All Late Onset Genes (seed genes and non‐seed genes)

**Table S5**. Genes shared between LO and EO in the Top 1% of Disease Relevant Genes

**Table S6**. Results CTLA4 locus fine mapping using PAINTOR

## Data Availability

All prioritized genes for EO and LO myasthenia gravis disease are available as Supplementary Data. Gene expression data sets used in this study are already available from the NCBI Gene Expression Omnibus (accession code: GSE78840) and ArrayExpress (accession codes: E‐MTAB‐945, E‐MTAB‐2232, and E‐MTAB‐3536). All other data are available from the corresponding authors upon reasonable requests.
